# Allele distribution and genetic diversity of VNTR loci in *Salmonella enterica *serotype Enteritidis isolates from different sources

**DOI:** 10.1186/1471-2180-8-146

**Published:** 2008-09-15

**Authors:** Seongbeom Cho, Thomas S Whittam, David J Boxrud, Joanne M Bartkus, A Mahdi Saeed

**Affiliations:** 1 National Food Safety and Toxicology Center, Michigan State University, East Lansing, MI, USA; 2 Molecular Epidemiology Unit, Minnesota Department of Health, St. Paul, MN, USA; 3 Departments of Large Animal Clinical Sciences and Epidemiology, Michigan State University, East Lansing, MI, USA

## Abstract

**Background:**

*Salmonella enterica *serotype Enteritidis (*S*. Enteritidis) is a zoonotic pathogen, which can be found in many sources including animals and the environment. However, little is known about the molecular relatedness among *S*. Enteritidis isolates from different sources. We have applied multiple-locus variable number tandem repeat analysis (MLVA) to study the genetic diversity of *S*. Enteritidis isolates from human and non-human sources.

**Results:**

We identified 38 unique MLVA types using nine VNTR loci markers for discrimination between 145 *S*. Enteritidis isolates from different sources including humans (n = 41), chickens (n = 45), and eggs (n = 40). There were 20 distinct MLVA types identified from human isolates, 17 distinct MLVA types from chicken isolates, and 5 from egg isolates. We compared allele distribution and frequency for each VNTR marker and measured allelic polymorphism within each VNTR locus of *S*. Enteritidis isolates from the sources using Nei's diversity index (*D*). Differences in allele distribution and frequency were detected in most loci of study isolates. Different genetic diversity for certain loci was identified in isolates from different sources. The average of genetic diversity (*D*) was lower in egg isolates (0.16) compared to human (0.41) and chicken (0.30). However, for loci SE3, SE7, and SE9, human isolates showed significantly higher diversity than both chicken and egg isolates. Whereas for loci SE5 and SE10, chicken isolates had significantly higher diversity than both human and egg isolates. Minimum-spanning tree (MST) comprised one major cluster, a minor cluster, and four clonal expansions. MLVA application enabled a cluster analysis by the MST of the *S*. Enteritidis isolates by sources, which allows a great insight into the genetic relatedness and the possible flow of these organisms between different reservoirs and humans.

**Conclusion:**

Differences in allele distribution and genetic diversity of VNTR loci in *S*. Enteritidis isolates from different sources were found. Polymorphism in most of the VNTR loci was more frequent among human *S*. Enteritidis isolates than isolates from chickens or eggs. Therefore, VNTR profiles of *S*. Enteritidis isolates from a specific source should be further evaluated as potential markers in epidemiologic investigations to trace *S*. Enteritidis to their probable source.

## Background

*Salmonella *serotypes are estimated to cause 1.4 million cases, more than 500 deaths, and cause severe economic losses which approach from $0.5 to 2.3 billion per year in the United States [1]. *Salmonella enterica *serotype Enteritidis (*S*. Enteritidis) emerged during the last three decades, currently ranking 2^nd ^most common serotypes in the United States. Furthermore, *S*. Enteritidis is the most common serotype in Europe and other parts of the worlds [2,3].

Ecologically, *S*. Enteritidis is a zoonotic pathogen that is harbored by many reservoirs and is transmissible to humans largely through contaminated foods. Several epidemiologic studies indicated the important role of eggs and poultry meat as major vehicles in the transmission of the organisms to human consumers [3-5]. *S*. Enteritidis can contaminate eggs through transovarian transmission during egg development in the infected chickens [6,7].

Genetically, *S*. Enteritidis probably originated from an ancestral clone that led to the evolution of several minor clones based on a phylogenetic analyses of a large groups of isolates [8]. However, little is known about the molecular relatedness among *S*. Enteritidis isolates from different reservoirs (or sources). While some reports described the existence of specific molecular attributes among *S*. Enteritidis associated with outbreak cases, the homogeneity of the *S*. Enteritidis genome renders typing tools such as PFGE insufficient for molecular characterization to establish relatedness among isolates from cases and probable sources for infection [9,10].

PFGE and phage typing have been combined to characterize *S*. Enteritidis isolates from different sources [10,11]. Although phage typing is still commonly used for the epidemiologic investigation of *S*. Enteritidis infections worldwide, this method has several shortcomings including the occurrence of non-typeable strains and the possible phage conversion among *S*. Enteritidis isolates [12]. Therefore, more efficient subtyping methods may be needed to relate disease-causing pathogens to their probable sources.

We have recently described an optimized MLVA technique using a single multiplex PCR followed by multicolor capillary gel electrophoresis and demonstrated that it has a higher discriminatory power than PFGE and phage typing in limited samples [13]. In that report, we suggested that MLVA subtyping together with PFGE would enhance the effectiveness of epidemiologic investigation of *S*. Enteritidis infections. The utility of VNTR analysis in characterizing *Salmonella *Typhimurium isolates from human, pig, and poultry was reported. The most frequent alleles at each locus were compared and it was concluded that the VNTR analysis might be potentially used for source attribution. [14].

In the present report we have updated the MLVA system using two panels of multiplex PCR and analyzed larger number of *S*. Enteritidis from different sources, including humans, chickens, and eggs. The objective of this study was to characterize *S*. Enteritidis isolates from human and non-human sources by 1) comparing the allele distribution of VNTR loci, 2) comparing genetic diversity of VNTR loci, and 3) describing the relationship between VNTR profiles and sources of isolates.

## Results

### Distribution of MLVA types by phage types among *S*. Enteritidis from different sources

The 145 *S*. Enteritidis isolates from different sources included 14 different phage types and 38 different MLVA types (Additional file 1). There were 20 distinct MLVA types identified from human isolates, 17 distinct MLVA types from chicken isolates, 5 from egg isolates, and 8 from other sources The most common MLVA type among isolates from humans was "A0". It was found in 9 (22%) of 41 human isolates. MLVA type "A9" was the most common (20%) among 17 distinct MLVA types found in 45 chicken isolates. MLVA type A4 was the most common (53%) among 5 different MLVA types found in 40 egg isolates.

Characterization of the most common *S*. Enteritidis phage types including PT8 (n = 46), PT13a (n = 45), PT28 (n = 25), and PT4 (n = 10) by specific MLVA types resulted in the following subgroups: Out of 46 *S*. Enteritidis isolates belonging to PT8, 15 isolates (32.6%) were MLVA type "A0" (χ^2^, *p *< 0.001) and15 isolates (32.6%) were MLVA type "A4" (χ^2^, *p *= 0.003).

The isolates with type "A0" and PT8 were significantly associated with humans (Fisher's exact, *p *< 0.05) whereas isolates with type "A4" and PT8 was significantly associated with egg source (χ^2^, *p *< 0.0001).

No significant associations were found between PT13a and specific MLVA types. Among 25 isolates of PT28, 4 belonged to MLVA type "A9", and 5 belonged to MLVA type "AM" (Fisher's exact, *p *< 0.05). The isolates with type "A9" and PT28 were significantly associated with chickens (Fisher's exact, *p *< 0.05) whereas isolates with type "AM" and PT28 were significantly associated with eggs. (Fisher's exact, *p *< 0.005). Among 10 isolates of PT4, 4 belonged to MLVA type "A6" (Fisher's exact, *p *< 0.01).

### Tandem repeats and allele distribution of VNTR loci among *S*. Enteritidis isolates from different sources

Most of the VNTR loci markers had different allele distribution among isolates from human chicken, and egg sources (Table 1). Significant differences in allele frequency at VNTR locus SE1 were found in isolates from chickens and eggs compared to isolates from humans (human vs. chicken; human vs. egg, *p *< 0.01). Significant differences in allele distribution were observed at the following VNTR loci: locus SE2 from the isolates (human vs. chicken, *p *< 0.05; human vs. egg, *p *< 0.01), locus SE3 (human vs. chicken; *p *= 0.05; human vs. egg, Fisher's exact, *p *< 0.01), locus SE5 (human vs. egg, *p *< 0.05), locus SE7 (human vs. chicken; human vs. egg, *p *< 0.01), locus SE8 (human vs. chicken; chicken vs. egg, *p *< 0.01), and locus SE9 (human vs. chicken; human vs. egg, Fisher's exact, *p *< 0.01).

**Table 1:** Allele distribution of nine VNTR loci of *S*. Enteritidis isolates from different sources

VNTR	Allele^§^	Human (n = 41)		Chicken (n = 45)		Egg (n = 40)		Others^b ^(n = 19)		Total (n = 145)	
Loci		No. (%)	*D*^a^	No. (%)	*D*	No. (%)	*D*	No. (%)	*D*	No. (%)	*D*
				
SE1* ^,+^	4	1 (2.4)								1 (0.7)	
	5	16 (39.0)						5 (26.3)		21 (14.5)	
	6	23 (56.1)		37 (82.2)		34 (85.0)		11 (57.9)		105 (72.4)	
	7	1 (2.4)		3 (6.7)		6 (15.0)		3 (15.8)		13 (9.0)	
	8		0.55	5 (11.1)	0.31		0.26		0.60	5 (3.4)	0.45
SE2* ^,+^	Null			2 (4.4)						2 (1.4)	
	5	4 (9.8)								4 (2.8)	
	6	17 (41.5)		3 (6.7)		7 (17.5)		10 (52.6)		37 (25.5)	
	8	1 (2.4)		2 (4.4)				4 (21.1)		7 (4.8)	
	9	16 (39.0)		25 (55.6)		33 (82.5)		2 (10.5)		76 (52.4)	
	10	2 (4.9)		12 (26.7)				3 (15.8)		17 (11.7)	
	11	1 (2.4)	0.68	1 (2.2)	0.63		0.30		0.68	2 (1.4)	0.65

SE3* ^,+^	3	10 (24.4)		4 (8.9)				6 (31.6)		20 (13.8)	
	4	30 (73.2)		41(91.1)		40 (100)		13 (68.4)		124 (85.5)	
	5	1 (2.4)	0.41		0.17		0.00		0.46	1 (0.7)	0.25

SE5^+^	6							3 (15.8)		3 (2.1)	
	7	1 (2.4)								1 (0.7)	
	9			1 (2.2)				3 (15.8)		4 (2.8)	
	10	1 (2.4)								1 (0.7)	
	11	19 (46.3)		16 (35.6)		11 (27.5)		3 (15.8)		49 (33.8)	
	12	16 (39.0)		10 (22.2)		29 (72.5)		5 (26.3)		60 (41.4)	
	13	3 (7.3)		12 (26.7)				5 (26.3)		20 (13.8)	
	14			3 (6.7)						3 (2.1)	
	17	1 (2.4)								1 (0.7)	
	18		0.64	3 (6.7)	0.76		0.41		0.83	3 (2.1)	0.70

SE6	Null			2 (4.4)						2 (1.4)	
	11	41 (100)		42 (93.3)		40 (100)		19 (100)		142 (97.9)	
	12		0.00	1 (2.2)	0.13		0.00		0.00	1 (0.7)	0.04

SE7* ^,+^	Null			3 (6.7)						3 (2.1)	
	4			1 (2.2)						1 (0.7)	
	5	1 (2.4)								1 (0.7)	
	6	2 (4.9)								2 (1.4)	
	7	10 (24.4)						6 (31.6)		16 (11.0)	
	8	28 (68.3)	0.48	41 (91.1)	0.17	40 (100)	0.00	13 (68.4)	0.46	122 (84.1)	0.28

SE8* ^,#^	1	22 (53.7)		11 (24.4)		27 (67.5)		6 (31.6)		66 (45.5)	
	2	19 (46.3)	0.51	34 (75.6)	0.38	13 (32.5)	0.45	13 (68.4)	0.46	79 (54.5)	0.50

SE9* ^,+^	2	31 (75.6)		45 (100)		40 (100)		16 (84.2)		132 (91.0)	
	3	10 (24.4)	0.38		0.00		0.00	3 (15.8)	0.28	13 (9.0)	0.16

SE10	7			4 (8.9)						4 (2.8)	
	8	41 (100)	0.00	41 (91.1)	0.17	40 (100)	0.00	19 (100)	0.00	141 (97.2)	0.05

^§^: Allele numbers corresponding to the numbers of tandem repeats for each of the nine VNTR loci

^a^: Nei's index of diversity as 1 - Σ (allele frequency)^2^

^b^: denotes chicken farm environment (n = 6), mouse (n = 4), mink (n = 4), bovine (n = 2), mule deer (n = 1), sea lion (n = 1) sources

Null: No amplification of the allele

*: Statistically significant difference between human and chicken for each VNTR locus

^+^: Statistically significant difference between human and egg for each VNTR locus

^#^: Statistically significant difference between chicken and egg for each VNTR locus

### Genetic Diversity of VNTR loci markers among isolates from different sources

The genetic diversity based on Nei's index of diversity (*D*) for nine VNTR loci ranged from 0.05 to 0.70 (Median: 0.28, Mean: 0.34). VNTR loci SE5 and SE2 were identified to be the most polymorphic loci (diversity indices of 0.70 and 0.65, respectively, Table 1) while loci SE6 and SE10 were less polymorphic with indices of less than 0.1.

The genetic diversity ranged from 0 to 0.68 (Median: 0.48; Mean: 0.41) for human isolates, 0 to 0.76 (Median: 0.17; Mean: 0.30) for chicken isolates, 0 to 0.45 (Median: 0.0; Mean: 0.16) for egg isolates, and 0 to 0.83 (Median: 0.45; Mean: 0.42) for others (Table 1). There were significant differences in the means of the diversity values (*D*) for seven polymorphic loci between isolates from human and egg sources (*p *< 0.001) and for eight polymorphic loci between isolates from chicken and egg sources (*p *= 0.01) while differences in the means for nine polymorphic loci between human and chicken isolates were not significant (*p *= 0.16).

Genetic diversity for each locus was compared between isolates from different sources (Additional file 2). Diversity for loci SE1, SE3, SE7, SE8, and SE9 was significantly higher in human isolates than in chicken isolates (*p *< 0.05) while the diversity for loci SE5 and SE10 was significantly higher in chicken isolates than in human isolates (*p *< 0.05). The genetic diversity values (*D*) for loci SE1, SE2, SE3, SE5, SE7, and SE9 were significantly higher in human isolates than in egg isolates (*p *< 0.05). The genetic diversity values (*D*) for loci SE2, SE3, SE5, SE7, and SE10 were significantly higher in chicken isolates than in egg isolates (*p *< 0.05).

Overall genotype diversity (based on Simpson's index of diversity) was significantly higher in MLVA (0.92, 95% CI: 0.90–0.95) compared to phage typing (0.77, 95%CI: 0.73–0.81). The MLVA genotype diversity was significantly higher in isolates from humans (0.93, 95% CI: 0.88–0.97) and isolates from chickens (0.91, 95% CI: 0.87–0.95), compared to isolates from eggs (0.68, 95%CI: 0.55–0.80).

### MLVA clusters by Minimum-spanning tree (MST)

**Figure 1:**
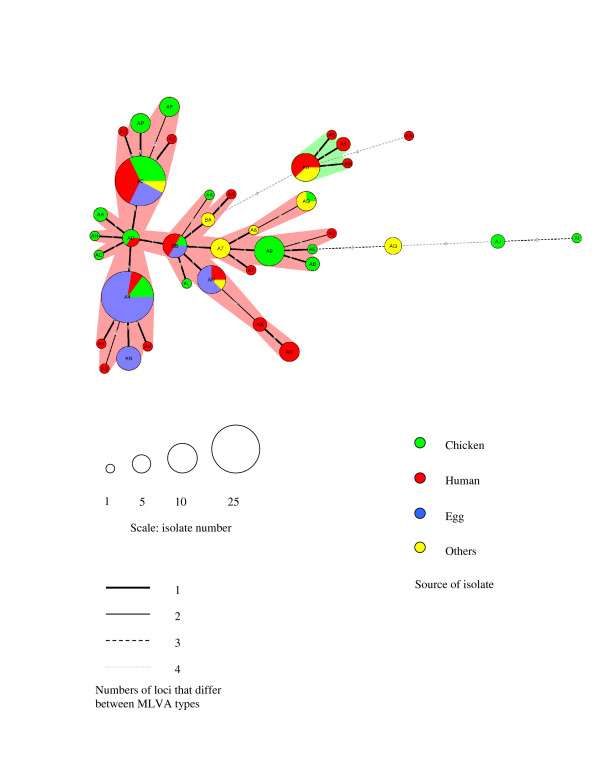
**Minimum-spanning tree of MLVA**. Each MLVA type is indicated by one node or branch tip, displayed as circles that are connected by branches of minimum-spanning tree. A two-letter code within each circle uniquely identifies each MLVA type, which is coded in a combination of the first letter ("A" or "B") and the second letter (any of alphabetical "A-Z" or a numerical "1–9 "). Clonal complexes were created based on maximum neighbor distance of changes at two loci and minimum size of two types. The length of the branches represents genetic distances (changes in loci) between two neighboring types. The sizes of the different color-circles depend on their population size. Wedges in circles indicate the proportion of isolates from respective sources with a particular MLVA type. In case of equivalent solutions in terms of calculated distance, the highest number of single locus variants (SLVs; in case two types have an equal distance to a linkage position in the tree, the type that has the highest number of SLVs is linked first) associated was used as the priority rule for linking types in the tree.

Minimum-spanning tree (MST) was created based on 38 distinct MLVA types of 145 isolates from four different sources as described in Figure 1. The MST yielded a major cluster, a minor cluster, and four clonal expansions from the clusters. Many source-specific clones were identified. In the major cluster, clone A4 primarily consisted of isolates from eggs [21/27 (78%)] and clone A9 comprised only isolates from chickens [9/9 (100%)]. A big clone A0 in the major cluster includes isolates from all four sources. A small cluster, which is genetically distant from the major cluster, consisted mostly of isolates from humans.

**Figure 2:**
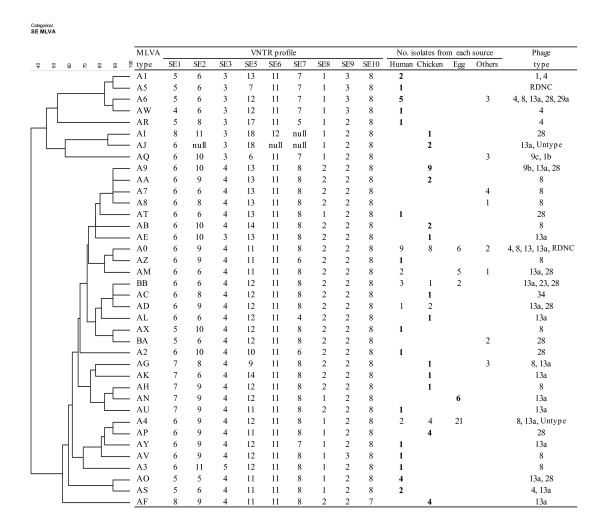
**Distribution of MLVA type with sources of isolates and VNTR profiles**. The categorical coefficients and UPGMA (unweighted pair group method with arithmetic averages) were used to generate a dendrogram. Null: No amplification of the allele. Bold type represents specific sources of *S*. Enteritidis (human, chicken, or egg).

Source-specific MLVA type is defined as a single MLVA type of *S*. Enteritidis isolate from a specific source. Of 38 MLVA types, 31 (81.6%) were source-specific and the other 7 MLVA types (18.4%) were associated with isolates from multiple sources. Among a total of 126 isolates from human, chicken, and egg sources, 60 isolates were classified into 29 source-specific MLVA types (Figure 2). Twenty-four (58.5 %) of 41 human *S*. Enteritidis isolates belonged to 15 source-specific MLVA types. The other 17 human isolates belonged to 5 MLVA types that were identified among isolates from non-human sources. However, there was no significant difference in the proportion of isolates belonging to specific MLVA types from humans compared to chickens and eggs (χ^2^, *p *= 0.09). Among the 45 chicken *S*. Enteritidis isolates, 30 (66.7%) isolates were classified into 13 source-specific MLVA types. These specific MLVA types were significantly associated with isolates from chickens compared to isolates from humans and eggs (χ^2^, *p *= 0.001). Six (15%) of 40 egg isolates belonged to one egg-specific MLVA type. Therefore, these findings suggest that specific MLVA types are more commonly associated with isolates from humans and chickens than isolates from eggs.

## Discussion

*Salmonella *Enteritidis circulates among several animal reservoirs particularly poultry, other animals and the environment. Human infections are frequently acquired from consumption of contaminated eggs and poultry meat as well as foods contaminated with the organism.

Prevention and control of the disease caused by *S*. Enteritidis in human and animal populations require sensitive and specific molecular epidemiologic tools. However, molecular relatedness among *S*. Enteritidis isolates from different reservoirs is not well studied.

Liebana et al [10] used genetic fingerprinting methods (ribotyping, PFGE, and plasmid profiling) for the assessment of diversity within *S*. Enteritidis isolates from poultry farms. They concluded that a single typing method was not sufficient for discrimination and that a more sensitive method is needed to discriminate between strains from the different geographical and animal origins [10].

Multi-locus variable number tandem repeat analysis (MLVA), which has been recently developed for subtyping of *S*. Enteritidis isolates from human sporadic cases, showed high epidemiological concordance in outbreak strains [15].

We have recently optimized and evaluated MLVA using a multiplex PCR and demonstrated sufficient allelic variation that subdivided the *S*. Enteritidis strains from human and non-human sources into numerous multilocus genotypes that constituted major clonal groups. Therefore, MLVA with high discriminatory power may be used to enhance the effectiveness of molecular epidemiologic investigation of *S*. Enteritidis infections [13].

In this study, we identified different allele distribution at most VNTR loci among *S*. Enteritidis isolates from different sources which suggests that patterns of allele distribution at some of the loci might be unique to isolates from specific sources. Therefore, the VNTR loci of unique allele distribution pattern can be used as potential markers for source tracking in the investigation of sporadic or outbreak cases.

Among the nine loci, SE2 and SE5 had a wide range of alleles per locus (higher variation of tandem repeats numbers) and their genetic diversity values (*D*) were also higher than other loci, resolving more frequent variation of these loci among isolates from humans and chickens than isolates from eggs (Table 1). Since these two loci may be hyper-mutatable, these findings are in support of the possible role of reservoir on the mutational rate or genetic variation of the *S*. Enteritidis isolates.

Variation in the number of repeat sequences at a given locus or sequence heterogeneity among individual units may be due to slipped-strand mispairing (SSM) which can occur in combination with inadequate DNA mismatch repair pathways during replication [16,17]. This instability can occur at a frequency of 10^-4 ^event per bacterial cell division and allows for a high frequency of genetic switching. Bacteria can use this random event to adapt their genetic repertoire in response to selective environmental pressure [17].

In this study, bacterial-host interaction could have contributed to broad genetic diversity of *S*. Enteritidis isolates from humans and chickens as compared to isolates from eggs that had significantly lesser genetic diversity. This may be explained in part by the fact that egg isolates represent lineage of specific clones of *S*. Enteritidis that are capable of causing transovarian transmission in the laying hens. A recent study suggested that single-nucleotide polymorphisms (SNPs) occurring in the genomic fragment of *S. enterica *was linked to genetic drift within *S*. Enteritidis that is associated with egg contamination [18]. Furthermore, a differentially regulated gene that is responsible for persistence or survival of the *S*. Enteritidis in egg albumin was identified [19]. Whether SNPs variations or the differentially regulated genes have a modulating effect on the VNTR diversity in isolates from eggs, more focused investigation including virulence profiling and VNTR analyses of isolates from different sources may be needed. Additionally, it is conceivable that humans may be more likely exposed to multiple strains of *S*. Enteritidis due perhaps to frequent travel or consumption of contaminated foods that may be originated from diverse locations or sources [15]. Therefore, it is conceivable that multiple infection sources and the high multiplication rate of *S*. Enteritidis in humans and chickens could have resulted in higher genetic diversity of these isolates compared to egg isolates in which the lower genetic diversity may be due to their clonal selection as invasive strains and their low multiplication inside eggs.

Stability of VNTR markers for *S*. Enteritidis has been previously reported [15] and in another study, VNTR markers for *S*. Typhimurium (including STTR3 and STTR5) were found to be stable during the course of outbreak [20]. Additionally, we have demonstrated the stability of VNTR loci in S. Enteritidis before and during experimental infection of a group of egg laying hens. VNTR profiles were studied on isolates from internal organs from a subgroup of the infected birds at two week-intervals during the one months experiment (unpublished data). Therefore, the stability of the VNTR markers in this study has been well documented.

We have included in this study, groups of isolates from human and nonhuman sources within the same time frame (1990s) for comparison using MLVA. Application of the same MLVA protocol on recent isolates from 245 human clinical cases of *Salmonella *Enteritidis that took place (between 2000–2007) produced similar useful MLVA-based groups (to be published elsewhere).

In comparing isolates from different sources for genetic diversity for each locus, different allelic diversity for certain loci was identified in isolates from different sources; human isolates showed significantly higher diversity in SE3, SE7, and SE9 loci than both chicken and egg isolates. Chicken isolates at loci SE5 and SE10 loci had significantly higher diversity than both human and egg isolates. These data may suggest an important role for the host in the genetic variation that can be encountered among *S*. Enteritidis isolates from different sources.

In this study, MLVA application enabled the MST cluster analysis of the *S*. Enteritidis isolates by source allowing a great insight into the possible flow of these organisms between different reservoirs and humans as the most important accidental host. The clusters show a close relatedness among egg isolates. The isolates in the minor cluster appeared to be genetically far distant from isolates in the major cluster. A minor cluster consists mostly of PT4-like isolates (PT4 and PT1) whereas most PT8-like isolates (including PT8, PT13a, and PT23) belonged to the major cluster. Most of the PT4 isolates from humans clustered together in the upper part of the dendrogram (Figure 2). These results are consistent with previous studies in which two separate lineages of serotype Enteritidis phage types were suggested based on difference in the LPS [21] and based on presence of a subset of phage regions [22].

For epidemiologic purposes, MLVA subtyping can be promising in that it is of high discriminatory power, reproducibility, is less labor-intensive than PFGE analysis, more easy to interpret and enables comparison of data between laboratories [23].

One of the advantages of MLVA over PFGE is that the variation that is resolved can be interpreted as allelic variation at specific chromosomal loci, thus opening the door for population genetic analyses and phylogenetic inference. Moreover, from a foodborne surveillance perspective, MLVA is more discriminatory than PFGE for *S*. Enteritidis strains and provides better epidemiological concordance [15]. Due to the ease and practical comparison of the profiles, MLVA can be used as a powerful subtyping tool for *S*. Enteritidis isolates in addition to current methods used to report molecular types of foodborne pathogens to central laboratories.

## Conclusion

We found that there are differences in allele distribution and genetic diversity of VNTR loci in *S*. Enteritidis isolates from different sources. Polymorphism in most of the VNTR loci was more frequent among human *S*. Enteritidis isolates than isolates from chickens or eggs. Multiple infection sources and rate of multiplication of *S*. Enteritidis in humans and chickens may lead to a higher genetic diversity whereas isolates from contaminated eggs have lower genetic diversity due to the fact that they may represent a select invasive clones of the organisms associated with a low multiplication inside the eggs. Therefore, VNTR profiles of *S*. Enteritidis isolates from a specific source should be further evaluated as potential markers for epidemiologic studies for tracing *S*. Enteritidis to their probable source.

## Methods

### Bacterial strains and sources

A total of 145 strains of *S*. Enteritidis (Additional file 3) were selected from a previously reported source pool (n = 1,273) of *S*. Enteritidis that were preserved at -80°C in Tryptic Soy Broth with 15 % glycerol [8]. Three main sources of strains consisted of humans (clinical isolates from human patients, n = 41), eggs (recovered from the internal contents of chicken eggs, n = 40), and chickens (cecal isolates representing the diversity of intestinally carried *S*. Enteritidis from chickens, n = 45). Most of the *S*. Enteritidis strains from human sources were isolated between 1990 and 1999 from sporadic cases of human *S*. Enteritidis infections in Indiana based on a collaborative effort with Indiana State Department of Health. Whereas, the egg isolates were from surveys we conducted in Pennsylvania and Indiana, two of the top five egg producing states in the nation during 1991–1994. Most of the chicken isolates were from cecal samples we cultured, as USDA-contractor for the National Spent Hen Surveys (1991–1995) to process the cecal samples from spent hens from 13 poultry farms in the Midwest [24].

Isolates from other sources included: mouse (n = 4), mink (n = 4), bovine (n = 2), mule deer (n = 1), sea lion (n = 1), canine (n = 1), and chicken farm environment (n = 6). The 145 strains were selected to make a representative sample collection for this study using selection by random digit numbers and using exclusion by consideration of phenotypic (phage type and attachment & invasiveness pattern to Hep2 cell), genotypic characteristics (MLEE), and year of isolation.

Purity of each isolate was confirmed with biochemical and serological testing and phage typing. Human *S*. Enteritidis isolates were phage typed at Centers for Disease Control and Prevention and isolates from animal sources were phage typed at the National Veterinary Service Laboratory (NVSL, Ames, IA) using phage typing scheme described by Ward et al (1987)[25].

### DNA isolation and multiplex PCR

DNA was extracted from each strain grown on tryptic soy agar plates overnight and then prepared as previously described [13]. Nine VNTR loci were amplified with two reaction sets of multiple primer mix (set A containing primer SE1, SE3, SE8, and SE10; set B containing primer SE2, SE5, SE6, SE7, and SE9) using fluorescently labeled forward primers (Sigma-Proligo, Boulder, CO) and non-labeled reverse primers (Integrated DNA Technologies, Coralville, IA) as described in Table 2. Tandem repeats sequences and their primers were described previously [13,15]. Primer sets for loci SE6 and SE10 were redesigned in this study to remove non-specific PCR amplicons when multiplex PCR is applied.

**Table 2:** VNTR loci on the genome of *S*. Enteritidis

Locus (alias)	Dye labeled	Primer sequences (5'-3')	PCR set	Conc. (uM)	Location (size)^b^; gene	Repeats size (bp)	No. repeats^c^	Reference
SE1	D3	F – AGACGTGGCAAGGAACAGTAG			LK5 Contig 1680_10.15			
		R – CCAGCCATCCATACCAAGAC	A	0.10	283–549 (267 bp)	7	5	[15]
SE3	D2	F – CAACAAAACAACAGCAGCAT			LK5 Contig 1921_10.15			
		R – GGGAAACGGTAATCAGAAAGT	A	0.10	537–856 (320 bp)	12	4	[15]
SE8	D2	F – TTGCCGCATAGCAGCAGAAGT			PT4			
		R – GCCTGAACACGCTTTTTAATAGGCT	A	0.15	2812703–2813171 (469 bp)	87	1	[15]
SE10^a^(STTR1)	D4	F – GCTGAAGAAGCGGCAAAAC			PT4			
		R – GTACCGCTATCTTTCGATGGC	A	0.05	774231–774760 (530 bp); SEN0697	45	8	[15,27]
SE2	D4	F – CTTCGGATTATACCTGGATTG			LK5 Contig 1930_10.15			
		R – TGGACGGAGGCGATAG	B	0.05	906–1106 (201 bp)	7	5	[15]
SE5 (STTR5, Sal16)	D2	F – CGGGAAACCACCATCAC			PT4			
		R – CAGGCCGAACAGCAGGAT	B	0.10	3073216–3073427 (212 bp); SEN2867	6	12	[15,27,28]
SE6^a^(STTR3, 3629542)	D3	F – CGGTGGCGGAGATTCTAATCA			PT4			
		R – ACGCCGTTGCTGAAGGTAAT	B	0.10	3510975–3511412 (438 bp); SEN3305	33	11	[15,27,29]
SE7	D4	F – CCGACCCAATAAGGAG			LK5 Contig 1168_10.15			
		R – CTTACCGTTGGTAGTTTGTTA	B	0.03	323–867 (545 bp)	61	8	[13,15]
SE9	D2	F – CGTAGCCAATCAGATTCATCCCGCG			PT4			
		R – TTTGAAACGGGGTGTGGCGCTG	B	0.10	533132–533460 (329 bp); SEN0475	9	3	[15]

^a^: Primer sets were redesigned in this study to remove non-specific PCR amplicons when multiplex PCR is applied.

Repeats sequences for locus SE6: TCGTCATCGCCGCTATCGTCGGGCGGGGTTACA

Repeats sequences for locus SE10: TTCGGCATCCGCTTTCTTCTTCGCGTCCGCCGCCGCTTTCGCCGC

^b^: The locations were based on the genome sequences of *Salmonella enterica *serovar Enteritidis LK5  and PT4  strains

^c^: Alignment parameters chosen to weight for match, mismatch and indels were 2.3.5 or 2.5.5 which is more permissive than other options . Repeat numbers for locus SE7 were counted manually due to imperfect repeat sequences. Repeat numbers for all loci are rounded to the nearest integer. For example, repeat number at locus SE7 in LK5 strain was rounded from 7.5 to 8.

A master mix was made with components for 15 μl reactions containing 7.5 μl of 2× Qiagen multiplex PCR Master mix, 1.8 μl of MgCl_2 _(25 mM; in a reaction set B), 1.5 μl of 10× primer mix (0.3–1.0 μM per each primer), 3 μl of the dilute DNA template, and RNase-free water to a volume of 15 μl. Samples were put on a GeneAmp PCR system 9700 (Applied Biosystems). The PCR conditions were 95°C for 15 min of pre-denature, then 35 cycles of 94°C for 30 s, 58°C for 90 s, 72°C for 90 s followed by a final elongation of 60°C for 30 min. The fragment size for each locus was determined by multicolor capillary gel separation as described in a previous study [13].

### VNTR analysis

Each locus for an *S*. Enteritidis isolate was assigned an allele score based on the fragment size. The allele scores were converted into repeats numbers of the nine loci and entered into BioNumerics software (Applied-Maths, St-Martens-Latem, Belgium) as character data for cluster analysis. Minimum-spanning tree and dendrogram were generated using the categorical coefficient of the software (version 4.61) as shown in Figure 1 and 2. This categorical parameter implies that the same weight is given to any multistate character at each locus, whatever the repeat number is. Hypothetical types (missing links) were introduced as branches of the MST, causing the total spanning of the tree to decrease significantly. In case of equivalent solutions in terms of calculated distance, the highest number of single locus variants was used as the priority rule for linking types in the tree as previously described [13].

### DNA sequencing

To verify the results from the Multi-Locus variable Number Tandem Repeat Analysis (MLVA) of *S*. Enteritidis, the copy number variation of tandem repeats for distinct alleles at all nine VNTR loci were analyzed. At least two different *S*. Enteritidis strains representing the same allele at each of nine VNTR loci were selected from different clusters of MLVA for sequencing. Sequence alignments were created using SeqMan (DNASTAR, Madison, WI) and the numbers of tandem repeats sequence for each locus were measured using Tandem Repeats Finder software (accessible at ) [26]. The copy numbers were rounded to the nearest integer (For example, 7.5 is rounded up to 8.0) and entered into the VNTR profiles.

### Genetic diversity

Nei's diversity index (*D*) was calculated for the measurement of genetic (allelic) diversity at each VNTR locus as 1 - Σ(allele frequency)^2^. The diversity indices were classified into four source-based groups: humans, chickens, eggs, and others (isolates from environment and other animals) to see if source-specific VNTR loci exist.

Simpson's index of diversity and its confidence interval were calculated to measure genotype diversity among isolates from different sources and the discriminatory power between MLVA subtyping and phage typing as previously stated [13].

### Statistical analysis

All statistical analyses for comparisons were performed using PC SAS system for Windows version 9.1 (SAS Institute, Cary, NC). Chi-square analysis or Fisher's exact test were performed to test for an association of dichotomous tabular data using PROC FREQ procedure while t-test was performed to compare the difference in the means of the polymorphic loci from groups of isolates using PROC TTEST. Comparisons with *p*-values < 0.05 were considered statistically significant.

## Authors' contributions

SC designed the study, carried out most of the experiment assays and data analyses, and led the writing of the manuscript. TSW contributed to the study design and final data analyses. DJB contributed to the experimental design and evaluation of results. JMB participated in the data evaluation and coordination. AMS oversaw the project, provided the bacterial isolates, and helped in completion of the manuscript. All authors read and approved the final manuscript.

## Supplementary Material

Additional file 1 Supplementary Figure S1. Distribution of Phage type and MLVA type among isolates from different sources. The common phage types among human isolates were PT8 (30%), PT13a (25%), PT28 (20%), and PT4 (17%). The common phage types among chicken isolates were PT13a (43%), PT8 (27%), and PT28 (22%). The common phage types among egg isolates were PT8 (42%), PT13a (30%), and PT28 (13%), and 15% of the isolates were untypable. Click here for file

Additional file 2 Supplementary Figure S2. Genetic diversity for 9 VNTR loci among human, chicken, and egg sources. Standard error bars are described in each histogram. >*: Significant difference between humans and chickens (higher in humans at loci SE1, SE3, SE7, SE8, and SE9; higher in chicken isolates at loci SE5 and SE10). ^+^: Significant difference between humans and eggs (higher in human isolates than egg isolates at loci SE1, SE2, SE3, SE5, SE7, and SE9). ^#^: Significant difference between chickens and eggs (higher in chicken isolates than egg isolates at loci SE2, SE3, SE5, SE7, and SE10) Click here for file

Additional file 3 Supplementary Table S3. Bacterial isolates with source information and VNTR profiles Click here for file
