# Supplementary material for: Allele distribution and genetic diversity of VNTR loci in Salmonella enterica serotype Enteritidis isolates from different sources
Source: BMC Microbiol. 2008 Sep 15;8:146. doi: 10.1186/1471-2180-8-146 (PMC2561042; doi:10.1186/1471-2180-8-146)
Supplement: Additional file 1 — Supplementary Figure S1. Distribution of Phage type and MLVA type among isolates from different sources. The common phage types among human isolates were PT8 (30%), PT13a (25%), PT28 (20%), and PT4 (17%). The common phage types among chicken isolates were PT13a (43%), PT8 (27%), and PT28 (22%). The common phage types among egg isolates were PT8 (42%), PT13a (30%), and PT28 (13%), and 15% of the isolates were untypable. [file 1471-2180-8-146-S1.doc]

(a)

(b)
