# Supplementary material for: Allele distribution and genetic diversity of VNTR loci in Salmonella enterica serotype Enteritidis isolates from different sources
Source: BMC Microbiol. 2008 Sep 15;8:146. doi: 10.1186/1471-2180-8-146 (PMC2561042; doi:10.1186/1471-2180-8-146)
Supplement: Additional file 2 — Supplementary Figure S2. Genetic diversity for 9 VNTR loci among human, chicken, and egg sources. Standard error bars are described in each histogram. >*: Significant difference between humans and chickens (higher in humans at loci SE1, SE3, SE7, SE8, and SE9; higher in chicken isolates at loci SE5 and SE10). +: Significant difference between humans and eggs (higher in human isolates than egg isolates at loci SE1, SE2, SE3, SE5, SE7, and SE9). #: Significant difference between chickens and eggs (higher in chicken isolates than egg isolates at loci SE2, SE3, SE5, SE7, and SE10) [file 1471-2180-8-146-S2.doc]

*,+

+,#

*,+,#

*

*,+

*,#

*,+,#

*,+,#
